# Periodontal status, tooth loss and self-reported periodontal problems effects on oral impacts on daily performances, OIDP, in pregnant women in Uganda: a cross-sectional study

**DOI:** 10.1186/1477-7525-7-89

**Published:** 2009-10-14

**Authors:** Margaret N Wandera, Ingunn M Engebretsen, Charles M Rwenyonyi, James Tumwine, Anne N Åstrøm, the PROMISE-EBF Study Group

**Affiliations:** 1 Institute of Clinical Odontology, Faculty of Medicine and Dentistry, University of Bergen, Norway; 2 Department of Dentistry, Makerere University, Uganda; 3 Center for International Health, University of Bergen, Norway; 4 Department of Paediatrics & Child Health, School of Medicine, Makerere University, Kampala, Uganda

## Abstract

**Background:**

An important aim of antenatal care is to improve maternal health- and well being of which oral health is an important part. This study aimed to estimate the prevalence of oral impacts on daily performances (OIDP) during pregnancy, using a locally adapted OIDP inventory, and to document how periodontal status, tooth-loss and reported periodontal problems are related to oral impacts.

**Methods:**

Pregnant women at about 7 months gestational age who were members of a community based multi-center cluster randomized community trial: PROMISE EBF: *Safety and Efficacy of Exclusive Breast feeding in the Era of HIV in Sub-Saharan Africa*, were recruited in the district of Mbale, Eastern Uganda between January 2006 and June 2008. A total of 877 women (participation rate 877/886, 98%, mean age 25.6, sd 6.4) completed an interview and 713 (participation rate 713/886, 80.6%, mean age 25.5 sd 6.6) were examined clinically with respect to tooth-loss and according to the Community Periodontal Index, CPI.

**Results:**

Seven of the original 8 OIDP items were translated into the local language. Cronbach's alpha was 0.85 and 0.80 in urban and rural areas, respectively. The prevalence of oral impacts was 25% in the urban and 30% in the rural area. Corresponding estimates for CPI>0 were 63% and 68%. Adjusted ORs for having any oral impact were 1.1 (95% CI 0.7-1.7), 1.9 (95% CI 1.2-3.1), 1.7 (1.1-2.7) and 2.0 (0.9-4.4) if having respectively, CPI>0, at least one tooth lost, tooth loss in molars and tooth loss in molar-and anterior regions. The Adjusted ORs for any oral impact if reporting periodontal problems ranged from 2.7(95% CI 1.8-4.2) (bad breath) through 8.6(95% CI 5.6-12.9) (chewing problem) to 22.3 (95% CI 13.3-35.9) (toothache).

**Conclusion:**

A substantial proportion of pregnant women experienced oral impacts. The OIDP impacts were most and least substantial regarding functional- and social concerns, respectively. The OIDP varied systematically with tooth loss in the molar region, reported chewing-and periodontal problems. Pregnant women's oral health should be addressed through antenatal care programs in societies with limited access to regular dental care facilities.

## Background

During pregnancy, hormones alter immuno-responsiveness and inflammatory response mediators. This has been reported to cause oral problems, primarily gingivitis and periodontal infection [1,2]. Pregnancy gingivitis ranges from asymptomatic erythema to severe cases with pain and bleeding of the gingival tissue, affecting 30%-100% of pregnant women in industrialized countries [3-5]. The severity of gingival inflammation has proved to be higher during pregnancy than after delivery, although no significant changes occur in the amount of plaque [6]. Moreover, gingival bleeding during pregnancy has been found to be less influenced by the method of oral hygiene applied and to be worse during the second- compared to the third trimester of pregnancy [7]. Whereas some studies have reported no association between parity (i.e. number of children borne) and tooth-loss, others have confirmed that increased parity is related to having fewer numbers of teeth [1,8].

Periodontal diseases produce a wide range of clinical signs and symptoms, such as tooth loss, altered appearance, pain, bleeding, bad breath and impaired quality of life [9,10]. Loss of posterior occluding support has been associated with impaired chewing efficiency and inadequate nutrition [11]. Inefficient chewing might increase the likelihood of over-preparing food in an effort to make consumption possible, whilst in this process, loosing important nutrients [12]. Inadequate nutrition during pregnancy may lead to poor fetal growth which might implicate health problems occurring in later life. For example, poor nutrition during pregnancy may lead to interference with kidney development in fetus, which in turn will lead to raised blood pressure in adulthood [13]. Recently, Taylor and Borgnakke [14] concluded that self-reported periodontal disease might be valid for surveillance of periodontal disease burden and trends in populations in lieu of more costly clinical examinations. Studies continue to document oral symptoms indicative of poor periodontal health occurring in at least one third of pregnant women in several countries [2,5,15]. Furthermore, pregnancy has been characterized by low use of dental services in spite of frequently reported periodontal symptoms [16].

A better understanding of the social, psychological and functional consequences of periodontal disease and tooth loss during pregnancy would assist the planning and evaluation of dental care for pregnant women and thus address their needs and concerns. So far, few studies have focused on how periodontal diseases affect the quality of life of the general population. Studies of the psycho-social consequences of the oral condition in pregnant women are almost non-existent and yet in two recent studies, considering referred periodontal patients in Great Britain and young adults in Hong Kong, the hypothesis that periodontal health impacts on people's quality of life was confirmed [17,18].

Since the seminal work of Cohen and Jago [19], researchers have increasingly been concerned with the functional and social consequences of oral problems and a number of instruments have been developed to measure oral health related quality of life (OHRQoL). The oral impact on daily performances (OIDP) is one of such instruments [20], developed to measure oral impacts that seriously affect a person's daily life activities. It consists of 8 items that assess the impact of oral conditions on basic activities and behaviours that cover the physical, psychological, and social dimensions of daily living. When applied in the context of low-income countries, the OIDP has shown to be psychometrically acceptable among the adolescent-, young adult- and elderly populations in Tanzania and Uganda [21,22]. There are no previous studies on the oral health of pregnant women in Uganda and only one study has so far applied OIDP in the context of pregnant women from a low income country, Brazil [23].

Focusing on pregnant women at about 7 months gestational age, resident in Mbale district, Eastern Uganda, this study aimed to estimate the prevalence of OIDP, and examine the relationship of oral impacts with periodontal status, tooth loss and self-reported symptoms suggestive of periodontal disease. Furthermore, this study examined whether tooth loss influenced self reported problems of chewing common Ugandan foods, and assessed the relationship of self reported chewing problems with OIDP.

## Methods

### Study area

Participating women of the present study were members of a multicentre randomized community trial and birth cohort study ("Safety and efficacy of exclusive breast feeding (EBF) promotion in an African setting with high prevalence of HIV"- PROMISE EBF) conducted in Uganda and three other sub Saharan African countries - Burkina Faso, Zambia and South Africa. A district was selected as the intervention site with the randomization unit being 1-2 villages of on average 1000 inhabitants (35 infants per year given a birth rate of 3.5%).

### Study population

Pregnant women resident in twenty four villages selected for randomization in urban and rural areas of Mbale district, Eastern Uganda, were recruited consecutively by local community leaders into the Promise EBF study between January 2006 and June 2008. Urban villages were sited within Mbale municipality while rural villages were sited in Bunghoko sub-county. A total of 886 pregnant women were eligible to participate in interviews and oral clinical examination. This number satisfied a sample size of 800 pregnant women calculated for the oral sub-study, assuming a prevalence of tooth loss (i.e. at least one tooth lost) of 50%, a precision of 0.05 and a design effect of 2. As this study included several outcomes, the size of the sample was calculated separately for each of them and the largest sample size required was adopted. The procedures of recruitment and participation in the Promise EBF study are detailed in another publication [24]. Ethical Clearance was obtained from the Ethical board, Faculty of Medicine, Makerere University. Written consent was obtained from all participants in the study and verbal consent was obtained prior to each examination and interview.

### Measures

Structured interviews were designed with EpiHandy software to be used on handheld computers [25]. Interviews were conducted in face to face settings with participants at household level. The interview schedules were developed in English and translated into the local language of Lumasaaba. Oral health professionals reviewed the interview schedule for semantic, experiential and conceptual equivalence and sensitivity to culture and selection of appropriate words were considered. The interview schedules were piloted before administration. The conceptual model adapted from the model of Wilson and Cleary [26] linking indicators of oral diseases to their symptomatic-, functional- and disability consequences was applied to identify factors to consider as determinants of OHRQoL and to structure the multivariate analyses. The interviews covered questions on mother's health status, socio-demographic characteristics and perceived oral health status. *Self-reported periodontal problems *were assessed by asking respondents about their experience with bleeding gums, color change in gums, swollen gums, tooth decay, bad breath, bad taste toothache and pain in gums. Responses were categorized as no = 0 and yes = 1. *Self-reported chewing problems *were assessed by asking women whether or not they anticipated difficulties eating seven Ugandan food items (green banana, millet bread/maize meal, rice, cassava, meat, vegetables and fish) (responses were 0 = no, 1 = yes) The food items were identified through discussions with residents of the area prior to designing the interview. The seven food items were added into a chewing problem index (range 0-7) and dichotomized into 0 = no difficulties with chewing food items and 1 = difficulty with chewing at least one food item. *Oral disadvantage *or the psychosocial consequences of oral disease and tissue damage were measured broadly using seven of the original eight item OIDP inventory (i.e. During the previous 6 months - how often have problems with your teeth and mouth caused you any difficulty with; eating, speaking, cleaning teeth, smiling, sleeping, work performance and social contact). The OIDP item considering emotional stability was removed due to problems with translation into the local language and possible misinterpretation by the study group. Each frequency item was scored 0-3, where (0) never, (1) less than once a month, (2) once or twice a month up to once or twice a week, (3) 3-4 times a week or more often. Finally, the extent of oral impacts, OIDP-extent, (range 0-7) was calculated as a simple count score (OIDP SC); i.e. summing dichotomized frequency items in terms of (1) affected (including the original categories 1,2,3) and (0) not affected (including the original category 0). *Socio-demographics *were assessed in terms of place of residence, age, educational level, last dental visit, parity and months of pregnancy. Family wealth was assessed as an indicator of socio-economic status in accordance with a standard approach in equity analyses [27]. Household durable assets indicative of family wealth (e.g. bicycle, television, car, motor cycle) assessed as (1) available/in working condition, (2) not available/nor in working condition were analyzed with principle component analysis, PCA. The first component resulting from the analysis was used to divide households into four approximate quartiles of wealth status ranging from 1^st ^quartile (least poor) to 4^th ^quartile (most poor). The socio-demographic variables controlled for in the analyses, their coding and the number of subjects (%) according to categories in urban and rural residence are shown in Table 1.

**Table 1:** Socio-demographic indicators among pregnant women in urban and rural areas of Mbale district. (n = 877)

		**Urban**		**Rural**		**p-value**
		**%**	(n = 234)*	**%**	(n = 633)*	
Age:	≤ 20 yr	28.4	(63)	25.6	(158)	
	21-30 yr	59.0	(131)	51.9	(320)	
	31-45 yr	12.6	(28)	22.5	(139)	0.006
Education:	Low (< 4 yr)	14.5	(31)	21.6	(122)	
(number of years in school)	Medium (5- 8 yr)	55.6	(119)	65.2	(369)	
	High (>9 yr)	29.9	(64)	13.3	(75)	0.000
Household assets:	1^st ^quartile-most poor	12.0	(26)	21.4	(130)	
	2^nd ^quartile	22.7	(49)	40.7	(247)	
	3^rd ^quartile	18.1	(39)	20.4	(124)	
	4^th ^quartile - least poor	47.2	(102)	17.5	(106)	0.000
Marital status:	Not married	48.7	(109)	34.7	(217)	
	Married	51.3	(115)	65.3	(409)	0.000
Last dental visit:	less than 6 months ago	8.3	(17)	4.7	(28)	
	more than 6 months	28.9	(59)	22.5	(134)	
	never	62.7	(128)	72.8	(433)	0.016
Months of pregnancy:	seven or more	87.5	(196)	83.4	(497)	
	less than seven	12.5	(28)	16.6	(99)	0.160
Parity:	one or more	74.6	(167)	78.3	(490)	
	none	25.4	(57)	21.7	(136)	0.265

*The total number of the various categories do not add to 877 due to missing values

### Clinical oral examination

A trained and calibrated dentist (MW) carried out all clinical oral examinations under field conditions based on the World Health Organization (WHO) criteria [28], recording the data on a prepared record sheet. All fully erupted permanent teeth were scored, excluding third molars. Oral examinations were performed at house hold level with subjects seated, examiner using a headlamp as source of illumination, mouth mirror and a periodontal probe. Neither radiographic examination nor drying of teeth was performed. *Periodontal status was *assessed using a specially designed lightweight CPITN probe with a 0,5 mm ball tip with periodontal pockets were measured from the edge of the free gingiva to the bottom of the pocket. Using the epidemiological part of the CPITN, the Community Periodontal Index (CPI) [28,29] with 10 index teeth (17,16,11,26,27,47,46,31,36,37) and 6 sextants (17-14, 13-23, 24-27, 38-34, 33-43, 44-47) per individual, four indicators of periodontal status were applied. Only index teeth were examined and the criteria used were; healthy periodontal status (code 0), bleeding on probing observed (code 1), calculus detected during probing (code 2), pocket 4-5 mm (code 3) and pocket >5 mm (code 4). Each index tooth was scored on 2 sites (buccal and lingual) and each sextant was scored according to its highest CPI score. If no index tooth was present in a sextant, all the remaining teeth in that sextant were examined and the highest score is recorded as the score for that sextant. In accordance with the hierarchical assumption of the CPI index, teeth with score 3 were assumed positive with respect to bleeding and calculus whereas teeth with score 2 were assumed positive with respect to bleeding [30]. Prevalence of bleeding-, calculus and pocket sextants was assessed as the percentage of subjects affected, or percentage of subjects having at least one affected sextant. Prevalence of healthy sextants was assessed as the number of subjects having 6 healthy sextants. Severity of periodontal condition was assessed by the mean number of sextants having CPI code 0,1,2,3 and 4. Total CPI was also presented as the percentage distribution of dentate subjects according to highest score in the mouth. For analyses this total CPI score was dichotomized into CPI = 0 and CPI>0. *Tooth-loss *was recorded for all teeth except the third molars and in terms of loss of any tooth (1 = yes, 0 = no), at least 1 tooth lost in both anterior & premolar regions (1 = yes, 0 = no), at least one tooth lost in molar region only (1 = yes, 0 = no) and at least 1 tooth lost in both in anterior & molar regions (1 = yes, 0 = no).

### Reproducibility

Duplicate clinical examinations were carried out on 50 mothers considered to be representative of the study participants after a period of one month. Analysis performed on the duplicate examination recordings gave Kappa values of 0.91 for missing teeth. With respect to indicators of periodontal condition, kappa values ranged from 0.48 (CPI index tooth 11) to 0.85 (CPI index tooth 31). These figures indicate moderate to good intra examiner reliability according to WHO [28].

### Statistical analysis

Data was analyzed using SPSS version 15.0 (Chicago, IL, USA). Cross tabulation, chi square statistics and Univariate ANOVA were used to assess bivariate relationships. Logistic regression analyses were conducted with OIDP and chewing problems using the logit model and 95% Confidence intervals (CI) given for the odds ratios.

## Results

### Description of the study population

A total of 877 women (mean age 25.6, sd 6.4) completed interviews at about 7 months gestational age. Of the 877 participants, 713 (mean age 25 yr) underwent clinical oral examination. The total participation rate was 80%. Reasons for not participating in the clinical examination were difficulties to locate women, withdrawal of consent and death. A total of 26.7% versus 73.3% (n = 877) of the participants were resident in urban and rural areas of Mbale district. The majority (84.6%) were in or beyond their 7 month of gestation. Only 2.7% of the women confirmed to use of any kind of tobacco product. The frequency distribution of socio-demographic characteristics varied systematically with place of residence (Table 1). Urban women were younger, had higher level of education, were less poor according to the wealth index, more often unmarried and more often dental visitors compared to their rural counterparts. Mean number of missing teeth was 0.79 (sd = 1.2) in urban and 0.75 (sd = 1.3) in rural areas. The corresponding prevalence of tooth loss was 42.5% and 33.8%. A total of 37.0%, 4.4%, 58.6% and 1.7% urban residents had total CPI scores of 0, 1, 2 and 3. Corresponding figures among rural residents were 31.7%, 2.8%, 65.3% and 0.2%.

### Non response analyses

One hundred and sixty four out of the 877 interviewed women did not participate in the clinical examination. In order to analyze the possibility that selection bias occurred from this sample attrition, a comparison was made of the socio-demographic characteristics of participants and non-participants. This non-response analysis revealed less substantial differences between the two groups with the frequency distributions of age, education, household assets and parity being similar. However, 78% versus 68% (p < 0.05) of respectively non-respondents and respondents reported having never visited a dentist.

### Psychometric properties, prevalence and socio-demographic distribution of OIDP

Cronbach's alpha for the 7 OIDP items was 0.81 (0.85 in urban and 0.80 in rural area). A total of 15.8% (84) and 70.3% (166) of participants without and with any oral impact (OIDP > 0) were dissatisfied with their oral health condition. In both urban and rural areas impacts on eating were most prevalent (24.5% in urban and 24.4% in rural), followed by cleaning (19.3% in urban and 21.3% in rural) and sleeping (19.1% in urban and 17.2% in rural). Fifty-nine (25.5%) and 175 (30.6%) of respectively, urban and rural participants confirmed experience with at least one oral impact on daily performance (Table 2). Among women with impacts, 27.1% had one, 22.5% two and 8.9% had seven oral impacts. The prevalence of OIDP in the total sample was 30.7% and the age distribution was 25.0%, 32.3% and 32.9% (ns), in respectively ≤ 20 yr-, 21-30 yr- and 31-45 yr olds. Oral impacts was more frequently reported among women with several previous births (multiparous) compared to their counterparts that had not yet given birth (primiparous) (p < 0.05) and among recent dental attendees, compared to non-attendees (p < 0.001).

**Table 2:** Prevalence of oral impacts and the mean oral impacts on daily performance score in pregnant women according to urban and rural place of residence (n = 877).

	**Experience of OIDP in last 6 months**				**OIDP score**			
	**Urban area**		**Rural area**		**Urban area**		**Rural area**	

	**%**	**(n)**	**%**	**(n)**	**Mean**	**(sd)**	**Mean**	**(sd)**
Eating	24.5	(50)	24.4	(145)	0.4	(0.7)	0.4	(0.7)
Speaking	8.8	(18)	9.1	(54)	0.11	(0.4)	0.1	(0.4)
Cleaning	19.8	(40)	21.3	(126)	0.3	(0.8)	0.4	(0.8)
Sleeping	19.1	(38)	17.2	(101)	0.2	(0.4)	0.2	(0.5)
Smiling	6.4	(13)	5.1	(30)	0.07	(0.3)	0.06	(0.2)
Carry out work	12.7	(26)	11.9	(70)	0.2	(0.4)	0.1	(0.4)
Enjoy social contact	6.8	(14)	6.2	(37)	0.07	(0.2)	0.06	(0.3)
**Overall**	**25.5**	**(59)**	**30.6**	**(175)**	**1.3**	**(2.6)**	**1.4**	**(2.6)**

### OIDP, periodontal condition and tooth loss

Since OIDP did not vary systematically with place of residence (urban/rural), it's distribution according to clinical- and self-reported oral problems was reported for the sample as a whole. As shown in Table 3, impacts on eating discriminated between those with CPI score 0 and those with CPI score >0 (20.1% versus 27.7%, p < 0.05). Each OIDP frequency item varied systematically with tooth loss in the molar region only and with tooth loss in the molar & anterior regions. Binary logistic regression analyses adjusting for potential confounding variables revealed adjusted odds ratios, OR, for experiencing any oral impact (OIDP > 0) of 1.9, 1.7 and 2.0 if having respectively, any tooth loss, tooth loss in molar region only and tooth loss in the anterior and molar regions (Table 4). A statistically significant two-way interaction occurred between tooth loss in the molar region and age group. Stratified analyses revealed that the OR for having OIDP > 0 if having tooth loss in the molar region declined with increasing age and were 9.7 (95% CI 3.8-24.5), 2.6, (95% CI 1.6-4.2) and 0.8 (95% CI 0.4-1.9), for the age groups <20 yr, 21-30 yr and 31-45 yr, respectively.

**Table 3:** Frequency distribution of oral impacts in pregnant Ugandan women according to Community Periodontal index and missing teeth (n = 713)

	**CPI = 0**		**CPI ≥ 1**		**No loss of tooth in anterior & molar region**		**Loss of at least 1 tooth in both anterior &** **Molar region**		**No loss of molar teeth**		**Loss of at least 1 molar tooth**	
**Impact**	**%**	**(n)**	**%**	**(n)**	**%**	**(n)**	**%**	**(n)**	**%**	**(n)**	**%**	**(n)**

Eating	20.1	(44)	27.7	(125)*	23.6	(150)	55.9	(19)**	20.2	(99)	38.7	(70)**
Speaking	7.8	(17)	9.3	(42)	8.2	(52)	21.2	(7)**	4.9	(24)	19.3	(35)**
Cleaning	17.4	(38)	22.8	(102)	20.2	(128)	36.4	(12)*	18.3	(89)	28.5	(51)*
Sleeping	15.6	(34)	18.8	(83)	16.1	(101)	48.5	(16)**	13.5	(65)	29.2	(52)**
Smiling	5.0	(11)	5.6	(25)	4.6	(29)	20.6	(7)**	3.5	(17)	10.6	(19)*
Carry out work	12.3	(27)	12.7	(57)	11.6	(74)	31.3	(10)*	8.2	(40)	24.4	(44)**
Enjoy social contact	5.9	(13)	7.1	(32)	6.1	(39)	17.6	(6)*	4.3	(21)	13.3	(24)**

* p < 0.05, ** p < 0.01

**Table 4:** Relationship of clinical indicators and oral impacts on daily performances in pregnant Ugandan women (percentages of those who had impacts, n = 713)

**Clinical indicator** **[% (n)]**	**Having impacts**		**Odds ratio (95% CI)**	**P-value**
	**%**	**(n)**		
**CPI^§^**				
CPI = 0 [33.0 (235)]	27.5	(55)	1.1 (0.7-1.7)	0.571
CPI ≥ 1 [67.0 (478)]	32.9	(142)		
**Missing at least 1 tooth^±^**				
No [64.0 (456)]	23.9	(100)	1	
At least 1 [36.0 (257)]	44.6	(100)	1.9 (1.2-3.1)	0.004
**Missing at least 1 tooth in both anterior & premolar^±^**				
No [96.4 (687)]	31.5	(196)	1	
Yes [3.6 (26)]	19.0	(4)	0.6 (0.2-1.8)	0.402
**Missing at least 1 molar^±^**				
No [72.5 (517)]	25.4	(119)	1	
Yes [27.5 (196)]	46.6	(81)**	1.7 (1.1-2.7)	0.020
**Missing at least 1 tooth in both anterior and molar^±^**				
No [95.1 (678)]	30.1	(185)	1	
Yes [4.9 (35)]	51.7	(15)*	2.0 (0.9-4.4)	0.084

^§ ^Odds ratios and 95% CI adjusted for age, urban/rural residence, parity, last dental visit and missed teeth

^±^Odds ratios and 95% CI adjusted for age, urban/rural residence, parity

* p < 0.05, ** p < 0.01

### Association of OIDP with reported symptoms suggestive of periodontal disease

The most commonly reported periodontal symptom was bleeding gums (49.8%), followed in descending order by toothache (31.8%) and pain in gums (24.2%). All reported symptoms discriminated statistically significantly between women with and without oral impacts. After adjusting for potential confounding variables in binary logistic regression analyses, the ORs for reporting any impact ranged from 2.7 (95% CI 1.8-4.2) with respect to bad breath to 22.3 (95% CI 13.3-35.9) regarding toothache (Table 5). A statistically significant two-way interaction occurred for the terms (bleeding gums × age). Stratified analyses revealed that the ORs of having any impact if reporting bleeding gums were 7.2 (95% CI 3.4-15.1), 5.1 (95% CI 3.1-8.2) and 2.0 (95% CI 0.99-4.0) for the age groups <20 yr, 21-30 yr and 31-45 yr, respectively.

**Table 5:** Relationship between self reported periodontal problems, problem chewing and oral impacts on daily performances in pregnant Ugandan women (percentages of those who had impacts, n = 877)

**During last 6 months:** **[% (n)]**	**Having impacts**		**OR (95% CI)**	**p-value**
	**%**	**(n)**		
**Bleeding gums^§^**				
No [50.2 (405)]	16.2	(63)	1	
Yes [49.8 (402)]	45.4	(173)	4.6 (3.1-7.0)	0.000
**Color change in gums^§^**				
No [86.8 (700)]	25.2	(168)	1	
Yes [13.2 (106)]	67.3	(68)	6.6 (3.9-11.2)	0.000
**Swollen gums^§^**				
No [80.2 (646)]	23.7	(146)	1	
Yes [19.8 (159)]	59.6	(90)	3.9 (2.5-6.1)	0.000
**Tooth decay^§^**				
No [72.5 (585)]	18.6	(104)	1	
Yes [27.5 (222)]	63.2	(132)	5.7 (3.8-8.7)	0.000
**Bad breath^§^**				
No [83.3 (667)]	24.7	(158)	1	
Yes [16.7 (134)]	60.5	(75)	3.7 (2.3-6.0)	0.000
**Bad taste^§^**				
No [77.4 (622)]	25.2	(150)	1	
Yes [22.6 (182)]	49.4	(84)	2.7 (1.8-4.2)	0.000
**Toothache^§^**				
No [68.2 (549)]	10.2	(54)	1	
Yes [31.8 (256)]	76.1	(181)	22.3 (13.3-35.9)	0.000
**Pain in gums^§^**				
No [75.8 (611)]	16.8	(99)	1	
Yes [24.2 (195)]	76.1	(137)	12.0 (7.6-18.9)	0.000
**Having chew problem^§^**				
No [68.6 (547)]	16.4%	(84)	1	
Yes [31.4 (250)]	64.1%	(150)	8.6 (5.6-12.9)	0.000

^§ ^OR and 95% CI adjusted for age, parity, urban/rural residence, last dental visit and missed teeth

### Association of OIDP and tooth loss with reported chewing problems

The prevalence of problems with chewing common Ugandan foods ranged from 28.9% concerning meat to 3.5% concerning matooke (green banana). A total of 31.4% had problems chewing at least one common food item. As shown in Table 6, the adjusted OR for reporting problem chewing at least one common food item was 1.8 (95% CI 1.2-3.0) if having lost at least one tooth whereas adjusted OR for reporting any oral impact (OIDP > 0) if having problem chewing at least one common food was 8.6 (95% CI 5.6-12.9).

**Table 6:** Relationship between difficulties chewing food item and missing teeth in pregnant Ugandan women (percentages of those who had problems chewing at least one food item) (n = 713)

	**Chewing problem**		**OR (95% CI)**	**P-value**
	**Unadjusted**		**Adjusted**	
	**%**	**(n)**		
**Missing teeth**				
No	27.5	(118)	1	
At least 1	43.6	(103)**	1.8 (1.2-3.0)	0.000
**Missing ≥ 1 in both anterior & premolar^±^**				
No	33.3	(214)		
Yes	31.8	(7) ns		
**Missing molars^±^**				
No	29.5	(143)	1	
Yes	43.3	(78)**	1.5 (0.9-2.3)	0.070
**Missing ≥ 1 in both anterior & molars^±^**				
No	32.2	(203)	1	
Yes	52.9	(18)*	1.8 (0.9-3.7)	0.101

^±^Odds ratios and 95% CI adjusted for age, parity, urban/rural residence and last dental visit

* p < 0.05, ** p < 0.01

## Discussion

An important aim of antenatal care is to improve maternal health and well-being of which dental health constitutes an integral part [15]. The present study applied for the first time a translated into Lumasaaba version of the OIDP frequency inventory to a sample of pregnant women resident in urban and rural areas of Mbale region, Eastern Uganda. Although the OIDP inventory has previously shown to be applicable to adolescents and young adults in Uganda [22], the present study setting necessitated a reestablishment of its psychometric properties including evaluation of the validity of the inventory. When used in personal interviews with pregnant women at household level, the translated 7-item OIDP frequency questionnaire had psychometric properties similar to its original English version shown to be applicable among young people from the general population in Uganda and Tanzania [21,22]. Cultural issues such as languages might give rise to problems with validity. However, hypothesis regarding the construct validity of the 7-item OIDP instrument was confirmed in that the total OIDP scores varied systematically and in the expected direction with women's general oral health perceptions. Although no approach can guarantee cross-cultural equivalence, the Lumaasaba version of the OIDP seemed to preserve the overall concepts of the English version except with respect to one single item, problems with emotional stability that could not be translated satisfactorily. This item created problems of interpretation and was therefore removed from the inventory. Besides this, the translated frequency OIDP questionnaire did not differ from its original version in terms of sequence of questions, the Likert scale (4-points) and recall period (6 months) used.

About one quarter (25% and 30%) of the urban and rural women interviewed had experienced at least one oral impact on daily performances in the 6 months preceding the survey (Table 3). This estimate is lower than the prevalence of impacts identified among Ugandan (age range 13-19 yr) and Tanzanian (age range 19-25) adolescents/younger adults from the general population [21,22]. However, the prevalence of OIDP presented in this study is comparable to that (33%) obtained in a Brazilian study of pregnant low income women using the original eight item OIDP frequency inventory [23]. Consistent with previous studies across various populations and age groups, eating problems was the most frequently reported aspect of oral impacts both in urban and rural women [23,31]. Thus, the frequency distribution of impacts varied from 25.5% (urban) and 24.4% (rural) with respect to eating problems to 6.8% (urban) and 6.2% (rural) with respect to social aspects such as enjoying contact with people. The corresponding rates in the Brazilian study were 22.8% and 11% [23]. A direct comparison between the present results and those obtained among pregnant women in Brazil should be done with caution as it is hampered by the use of slightly different methodologies. In the Brazilian study questions on oral impacts were asked to women who confirmed oral pain, whereas in this study all participants completed the OIDP inventory independent of symptom status.

The present results demonstrate a strong association between the total OIDP score and some clinical indicators such as tooth loss, and no association with others, such as the total CPI score (Table 5). The lack of a significant relationship between OIDP total and CPI scores might be attributed to the low severity of periodontal condition observed in this sample of pregnant women, with only about 1-2% showing pocket depths of 4-5 mm (CPI score 3). In contrast, studies of dental attendees with severe periodontal disease have presented a significant relationship between periodontal disease and OHRQoL using the UK oral health related quality of life- and the Chinese short-form version of the OHIP instruments [17,18]. As shown in Table 4 and 5, tooth loss in the molar region was strongly related to the various OIDP items and to the OIDP total scores, despite the relatively low prevalence of OIDP and tooth loss presented. After adjusting for age, parity, urban/rural residency and last dental visit, women having lost at least one tooth and those having tooth loss in the molar region were 1.9 and 1.7 times more likely than their counterparts to report any OIDP. The relationship between OIDP and tooth loss involving the anterior region was in the expected direction but not statistically significant. Thus, dental appearance seems to be less important than dental functioning among pregnant women, particularly so in the younger age groups. This interpretation is supported by the higher prevalence of impacts related to function (eating, cleaning) than to appearance and social concerns (smiling and showing teeth). Similarly, the most frequently mentioned oral impacts reported by Brazilian pregnant women were functional in terms of problems with eating and cleaning teeth [23].

About three quarters of the participants had never visited a dentist (Table 1), indicating that they were at best non-regular attendees with less control or treatment of their oral condition. This is noteworthy as there is growing evidence supporting the importance of good oral health during pregnancy among other things to prevent adverse pregnancy outcomes [32-34]. The dental attendance patterns in terms of few regular dental visitors corroborates findings pertaining to pregnant women from other cultures [2,3,5,15,16] and is in line with those of young adults from the general East African populations [35]. Reports from the United States of America indicate that more than 50% of pregnant women did not receive dental care during their recent pregnancy [36]. In developed countries the belief of 'one tooth, one child' is widespread, meanwhile many oral health providers still consider pregnancy unsafe for dental procedures without the supporting evidence [1]. Limited access to oral health care in Uganda populations is generally due to the concentration of the few available services in urban areas and the low priority given to oral health services in the public resource allocation [37]. The low frequency of attendance in this Ugandan population might reflect limited availability of appropriate dental care and myths surrounding safety of dental care during pregnancy. Furthermore, it might be attributed to a low level of importance of oral diseases as perceived by pregnant women in this socio-cultural context for whom the prevailing levels of Malaria, HIV/AIDS, poverty, social crisis and weak health systems are much more severe.

Variation in the total OIDP score by *self-reported periodontal *symptoms was apparent even after controlling for possible confounding variables. The OIDP discriminated most strongly between women with- and without toothache (OR = 22.3) and pain in gums (OR = 12.0). This corroborates findings reported in pregnant Brazilian women and adds further support to the construct validity of the OIDP instrument in this particular social context [23]. Toothache, bleeding gums and change in gum color impacted OHRQoL most strongly in younger women and among women without missing teeth. These variations in the relationship between periodontal symptoms and OIDP might be attributed to differences in oral health related expectations and attitudes. Whereas about 10% and 60% and of the study participants had respectively 1 (bleeding on probing) and 2 (calculus) as their highest CPI scores, 49%, 13% and 24% reported bleeding gums, color change of gums and pain in gums, respectively. These rates of reported periodontal symptoms observed accords with those reported among UK pregnant women where about one third reported deterioration in either teeth or gums during pregnancy [15]. Underestimation of disease experience in self-reports of periodontal condition when compared to corresponding clinical measures as reported among pregnant women in Denmark was not observed in the present study [5]. Whether the generic OIDP inventory is sensitive to both clinically assessed and self reported periodontal health among pregnant women in Uganda is questionable and has to be investigated further in subsequent studies.

About one third had problems chewing any common Ugandan food (30%) and difficulties with chewing were most frequently reported in women with tooth loss in the molar region. Accordingly, Sarita et al [38] reported that subjects with severely reduced posterior occluding support were those most likely to have chewing complaints. The significant relationship of tooth loss with reported chewing problems and of reported chewing problems with oral quality of life supports what has been reported in previous studies considering the general adult population in East Africa [11].

## Conclusion

The impact of oral health on pregnant women's quality of life was assessed using a locally adapted 7-item-OIDP inventory. The OIDP prevalence showed impacts to be substantial regarding functional aspects, and less with respect to appearance and social concerns. The OIDP instrument demonstrated discriminative validity in identifying women with clinical evidence of tooth loss, but was less convincing in identifying women with clinically defined periodontal disease. Self reported periodontal symptoms as well as reported chewing problems showed significant relationships with OIDP. Intraoral changes that occur in pregnancy combined with limited access to regular dental care put pregnant women at risk for numerous oral impacts on their health and well being. This calls for improved oral health education and oral health care in Ugandan pregnant women. Oral health education might preferably be integrated into already existing antenatal health care programs. Oral health care professionals should be at the forefront advocating for resource mobilization to improve access to appropriate oral health care during pregnancy.

## Competing interests

The authors declare that they have no competing interests.

## Authors' contributions

All authors contributed to design of study.

MW: Principal investigator, collected data, statistical analyses and manuscript writing

ANÅ: Main supervisor, statistical analyses, and manuscript writing

IMSE: contributed to manuscript writing

CMR and JKT: supervised data collection and have been involved in revising manuscript

## Financial support

The study was part of the EU-funded project PROMISE-EBF (contract no INCO-CT 2004-003660, web ). It was also financially supported by Norwegian Research Council (project number 156744) funded project *Oral health in a global perspective*.
